# Flavin-dependent *N*-hydroxylating enzymes: distribution and application

**DOI:** 10.1007/s00253-020-10705-w

**Published:** 2020-06-05

**Authors:** Carolin Mügge, Thomas Heine, Alvaro Gomez Baraibar, Willem J. H. van Berkel, Caroline E. Paul, Dirk Tischler

**Affiliations:** 1 Microbial Biotechnology, Faculty of Biology and Biotechnology, Ruhr-Universität Bochum, Universitätsstr. 150, 44780 Bochum, Germany; 2 Environmental Microbiology, Faculty of Chemistry and Physics, TU Bergakademie Freiberg, Leipziger Str. 29, 09599 Freiberg, Germany; 3 Present Address: Rottendorf Pharma GmbH, Ostenfelder Str. 51-61, 59320 Ennigerloh, Germany; 4 Laboratory of Food Chemistry, Wageningen University & Research, Bornse Weilanden 9, 6708 WG Wageningen, The Netherlands; 5 Biocatalysis, Department of Biotechnology, Delft University of Technology, Van der Maasweg 9, HZ 2629 Delft, The Netherlands

**Keywords:** Biotransformation, *N*-Hydroxylases, Flavoproteins, Siderophores, Bioactive compounds, Biocatalysis, Monooxygenases, Phylogenetics

## Abstract

Amino groups derived from naturally abundant amino acids or (di)amines can be used as “shuttles” in nature for oxygen transfer to provide intermediates or products comprising N-O functional groups such as *N*-hydroxy, oxazine, isoxazolidine, nitro, nitrone, oxime, *C*-, *S*-, or *N*-nitroso, and azoxy units. To this end, molecular oxygen is activated by flavin, heme, or metal cofactor-containing enzymes and transferred to initially obtain *N*-hydroxy compounds, which can be further functionalized. In this review, we focus on flavin-dependent *N*-hydroxylating enzymes, which play a major role in the production of secondary metabolites, such as siderophores or antimicrobial agents. Flavoprotein monooxygenases of higher organisms (among others, in humans) can interact with nitrogen-bearing secondary metabolites or are relevant with respect to detoxification metabolism and are thus of importance to understand potential medical applications. Many enzymes that catalyze *N*-hydroxylation reactions have specific substrate scopes and others are rather relaxed. The subsequent conversion towards various N-O or N-N comprising molecules is also described. Overall, flavin-dependent *N*-hydroxylating enzymes can accept amines, diamines, amino acids, amino sugars, and amino aromatic compounds and thus provide access to versatile families of compounds containing the N-O motif. Natural roles as well as synthetic applications are highlighted.

**Table:** 

Key points *• N-O and N-N comprising natural and* (*semi*)*synthetic products are highlighted*. *• Flavin-based NMOs with respect to mechanism*, *structure*, *and phylogeny are reviewed*. *• Applications in natural product formation and synthetic approaches are provided*.

Key points

*• N-O and N-N comprising natural and* (*semi*)*synthetic products are highlighted*.

*• Flavin-based NMOs with respect to mechanism*, *structure*, *and phylogeny are reviewed*.

*• Applications in natural product formation and synthetic approaches are provided*.

**Graphical abstract:**
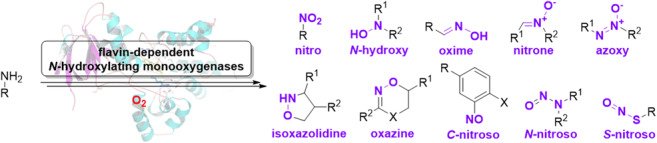
.

.

## Introduction

Nature provides access to numerous complex molecules with a variety of activities. The respective function is based on the molecular character of individual compounds and often shaped by their functional groups (Waldman et al. 2017; Davison and Sperry 2017). Among those, many comprise heteroatoms as N, O, S, and P or even a combination thereof. Because heteroatom-containing functional groups not vastly abundant in biological systems usually provide special functions, they have been moving into the focus of recent biotechnological research due to their potential for novel applications (Sulzbach and Kunjapur 2020). Herein, we will focus especially on N-O containing functional groups (mainly *N*-hydroxy; others are oxime, azoxy, oxazine, nitrone, *C*-nitroso, *N*-nitroso, *S*-nitroso, nitro, isoxazolidine), which are often found in secondary metabolites. Those natural products are interesting for many reasons, as they comprise antibiotics, bioactive agents, flavor and fragrance molecules, food additives, pharmaceutical precursors or products, siderophores and metallophores, enzyme inhibitors, and toxins, among others.

**Fig. 1:**
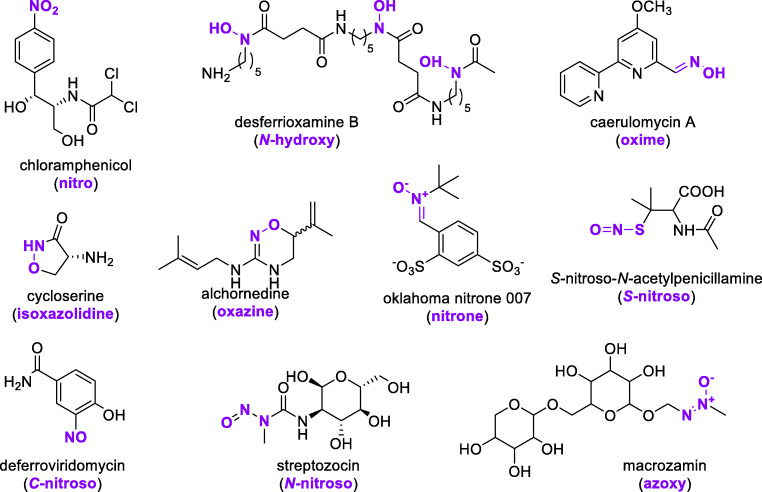
N-O functional groups in representative and already used naturally occurring or bio-produced compounds. The formation of N-N bonds can also be initially promoted by intermediate N-O bond formation, and thus, more complex structures can be obtained (vide infra). These structures are only representatives and often many derivatives occur due to natural diversity or synthetic modifications to provide more significant effects in desired fields

The formation of the various N-O linkages (Fig. 1) in organic molecules is often based on the conversion of soft nucleophilic amines by a form of activated (molecular) oxygen and combined with potential maturation reactions. Sometimes, overoxidation alone leads to a mixture of N-O functional groups in a compound. Nevertheless, a variety of enzymes have evolved to selectively activate oxygen to transfer it onto target compounds, e.g., onto amino groups. Here, especially flavin-dependent monooxygenases, heme-dependent P450 monooxygenases, peroxygenases, and other metal-dependent mono- and dioxygenases can be mentioned. Successive oxidation reactions might change the redox state of respective compounds to yield the final N-O or in other cases N-N functionality containing molecules.

In this review, we mainly focus on the properties of flavin-dependent *N*-hydroxylating monooxygenases (NMOs) and present recent findings about these enzymes since the last reviews six and more years ago (Olucha and Lamb 2011; Robinson and Sobrado 2012; Huijbers et al. 2014).

We first depict the catalytic mechanism and structural properties of NMOs, followed by recent advances and results from in-depth enzymology studies especially in the context of siderophore synthesis, where NMOs catalyze the *N*-hydroxylation of defined small substrates (i.e., diamino acids and diamines) at the beginning of biosynthetic pathways. Here, structural diversification typically happens in variable downstream conversions. Furthermore, we highlight NMOs that accept more complex substrates, as late diversification stages of complex molecules or involved in the metabolization of bioactive compounds. Lastly, we describe NMOs involved in the formation of N-N and N=N bonds and reveal which common biological pathways are used by microorganisms to produce this great structural diversity. Few NMOs are currently exploited for biotechnological applications, leaving the vast majority of known—but hitherto uncharacterized in terms of enzymatic performance—enzymes as potential research target aiming at the production of complex chemical compounds.

## Catalytic mechanism of flavin-dependent NMOs

Flavin-dependent NMOs (EC 1.14.13.x) produce numerous important products, but only a few family members have been thoroughly characterized to date. NMOs belong to the single-component group B flavoprotein monooxygenases (Fraaije and van Berkel 2006; van Berkel et al. 2006; Olucha and Lamb 2011; Huijbers et al. 2014). It needs to be mentioned that some related group B enzymes are also able to perform *N*-hydroxylations. Those enzymes are typically designated as FMOs, a term which has been introduced for flavin-containing monooxygenases being involved in mammalian detoxification processes (EC 1.14.13.8) (Ziegler 1988; van Berkel and Müller 1991; Ziegler 2002; van Berkel et al. 2006; Huijbers et al. 2014; Mascotti et al. 2015). Therefore, the abbreviation FMO is not synonymous to NMO but represents a specific subgroup of (mostly) eukaryotic group B flavin-containing enzymes that catalyze the monooxygenation of carbon-bound reactive heteroatoms including nitrogen, sulfur, phosphorous, selenium, and iodine (van Berkel et al. 2006). Also, the group of YUCCA and related enzymes from plants form a subgroup of group B flavin-containing monooxygenases and thus are related to type I BVMOs, NMOs, and FMOs (Huijbers et al. 2014).

**Fig. 2:**
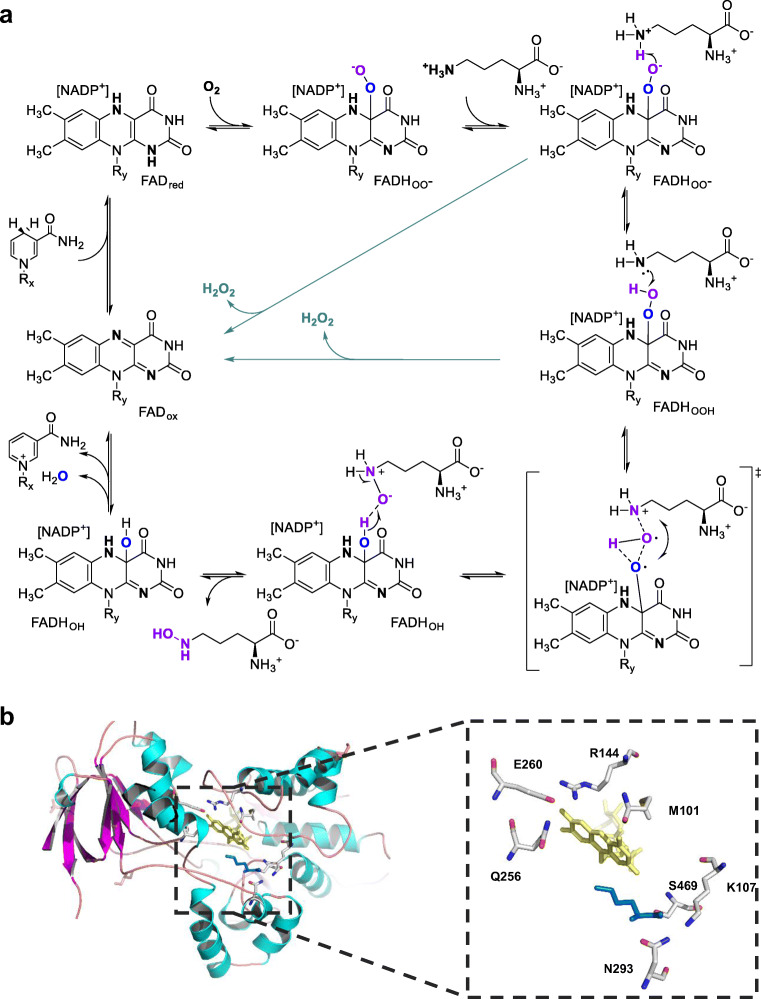
**a** Catalytic mechanism of flavin-dependent NMOs as reported for SidA and PvdA. The order of entry of oxygen and ornithine, and the ease of formation of FADH_OOH_ from FADH_OO-_ depends on the enzyme (for details see text). **b** Three-dimensional model of the crystal structure of SidA from *A. fumigatus* (PDB code: 4B69) (Franceschini et al. 2012). The SidA structure is colored according to secondary structural elements. The FAD cofactor is shown in yellow and the substrate ornithine in marine blue. A zoom into the active site indicates, next to the flavin and the substrate, several important amino acid residues

Members of group B flavoprotein monooxygenases contain two Rossmann-type dinucleotide binding domains to harbor the flavin adenine dinucleotide (FAD) and nicotinamide adenine dinucleotide (NAD(P)H) cofactors and keep the pyridine nucleotide bound during the oxidative half-reaction. In general, the mechanism of NMOs is similar to that of group B Baeyer-Villiger monooxygenases (BVMOs) (Ryerson et al. 1982; Sheng et al. 2001; Ballou and Entsch 2013) and can be described as follows (Fig. 2a) (Olucha and Lamb 2011; Bufkin and Sobrado 2017):

In its resting state, the enzyme harbors an oxidized FAD (FAD_ox_), as determined for SidA from *Aspergillus fumigatus* (Alfieri et al. 2008; Chocklett and Sobrado 2010; Romero et al. 2012a; Franceschini et al. 2012; Robinson et al. 2013; Robinson et al. 2014b) and PvdA from *Pseudomonas aeruginosa* (Meneely et al. 2009). Catalysis is initiated by the binding of NADPH to FAD_ox_. Then, FAD_ox_ is reduced to FADH^−^ (FAD_red_) (Fig. 2a). For both SidA and PvdA, this step is among the slowest in the overall catalysis cycle (Meneely et al. 2009; Mayfield et al. 2010; Romero et al. 2012a). Furthermore, PvdA is highly specific for NADPH (Meneely and Lamb 2007), whereas SidA accepts both coenzymes with a preference for NADPH (Chocklett and Sobrado 2010).

Generating the FAD_red_-NADP^+^ complex allows binding and activation of molecular oxygen to form the stable, long-lived C4a-hydroperoxflavin (FADH_OOH_, Fig. 2a) (Massey 1994). The rate of formation of this oxygenation species is moderately enhanced by the binding of ornithine (Frederick et al. 2011). In PvdA, the formation of FADH_OOH_ from C4a-peroxyflavin (FADH_OO_-) is dependent on substrate binding (Meneely et al. 2009), while in SidA, FADH_OOH_ immediately forms independent of substrate binding (van Berkel et al. 2011; Frederick et al. 2011; Romero et al. 2012b). Based on solvent kinetic isotope effects, density functional theory analysis, and the structural arrangement of the active site, it was argued that in SidA, the 2’OH of the ribose of NADP^+^ delivers the proton for the conversion of FADH_OO_- into FADH_OOH_ (Robinson et al. 2014b).

Formation of the ternary complex between FADH_OOH_, NADP^+^, and substrate allows the selective *N-*hydroxylation of the substrate (Fig. 2a). In the case of SidA and PvdA, ornithine is oxidized to *N*^*5*^-hydroxyornithine. During this step, hydroxy-FAD (FADH_OH_) is formed, which decays to FAD_ox_ and H_2_O. Finally, after the release of *N*^*6*^-hydroxyornithine and NADP^+^, FAD_ox_ is ready for the next cycle.

The stabilization of the reactive C4a-hydroperoxyflavin is crucial for this mechanism in order to prevent unproductive formation of hydrogen peroxide (designated as uncoupling) and wasting the reducing equivalents of NADPH. In the case of SidA and PvdA, which are both highly specific for the *N*^*5*^-hydroxylation of l-ornithine, uncoupling occurs with a range of substrate analogs, including among others d-ornithine, l-arginine, and l-lysine. Structural studies of PvdA indicated that an optimal orientation of the amino group of the substrate is required to prevent uncoupling (Olucha et al. 2011). The bound NADP^+^ plays a critical role here, because upon FAD reduction, its nicotinamide ring changes position, thereby creating a pocket for the formation and stabilization of the C4a-hydroperoxyflavin (Olucha et al. 2011).

The lysine hydroxylase MbsG from *Mycobacterium smegmatis* features a somewhat different kinetic mechanism (Robinson et al. 2014b). Here, lysine binds before the pyridine nucleotide, resulting in a substrate-NADPH-FAD_ox_ ternary complex. Substrate binding slightly decreases the rate of flavin reduction, but hardly influences the rate of the reaction with oxygen. Another difference between SidA and PvdA is that MbsG has a slight preference for NADH (Robinson and Sobrado 2012).

The crystal structure of PvdA (PDB code 3S5W) was the first NMO structure solved (Olucha et al. 2011). This structure of the ternary complex with bound l-ornithine and NADP(H) revealed that the active site is located at the interface of three domains. Next to the FAD- and NADPH-binding domains, the enzyme contains a small helical domain for binding the ornithine substrate. The PvdA structure also revealed that Arg240 and Ser286 were in H-bond distance of the 2′-phosphate group of the adenosine ribose in NADPH, thereby determining the coenzyme specificity.

Crystallographic analysis of the SidA tetramer allowed the three-dimensional structure determination of seven SidA-ligand complexes, including the binary and ternary complexes of FAD_ox_ and FAD_red_ bound to ornithine, lysine, arginine, and NADP^+^ (Franceschini et al. 2012). The structure of the enzyme-substrate complex as depicted in Fig. 2b is highly similar to the corresponding complex of PvdA (Olucha et al. 2011). For more details about the function of the active site residues highlighted in Fig. 2b, we refer to the original articles, which gave clues about the strict selectivity of both enzymes for l-ornithine, the essential role of NADP^+^ in stabilizing the C4a-hydroperoxyflavin, and the active site environment where the hydroxylation of the substrate takes place.

Site-directed mutagenesis of SidA was then used to investigate the predicted role of certain critical residues. Changing Ser257, involved in binding the pyrophosphate moiety of NADPH, to Ala revealed that this serine was important for the correct positioning of NADP^+^, thereby stabilizing the C4a-hydroperoxyflavin (Shirey et al. 2013). Replacement of Arg279, which interacts with the 2′-phosphate of NADPH, by Ala or Glu resulted in a different coenzyme specificity and strong uncoupling of hydroxylation. The R279A variant showed no coenzyme preference, while R279E preferred NADH as coenzyme. The fact that the changes in coenzyme preference were mainly due to changes in coenzyme binding strength corroborated the hypothesis that the positive charge at position 279 is crucial for the tight binding of NADPH (Robinson et al. 2014a).

Four residues involved in ornithine binding were individually changed to Ala (Robinson et al. 2015). The Lys107Ala variant lost its hydroxylation activity, indicating that the ionic interaction between Lys107 and the substrate carboxylate is essential for catalysis. Mutation of Asn293 and Ser469 to Ala strongly weakened the binding of ornithine. A similar effect was observed with the N323A variant. Besides interacting with the substrate, Asn323 also interacts with the nicotinamide ribose of NADPH. The crystal structure of the N323A variant complexed with ornithine and NADP^+^ revealed a disordered binding mode of the nicotinamide ribose group, while kinetic experiments showed that this mutated variant was much faster than wild-type SidA in reducing the flavin cofactor. The fact that Asn323 facilitates substrate binding at the expense of hindering flavin reduction clearly demonstrates the delicate balance of the enzyme-ligand interaction network in the SidA active site.

## NMOs involved in siderophore biosynthesis

**Fig. 3:**
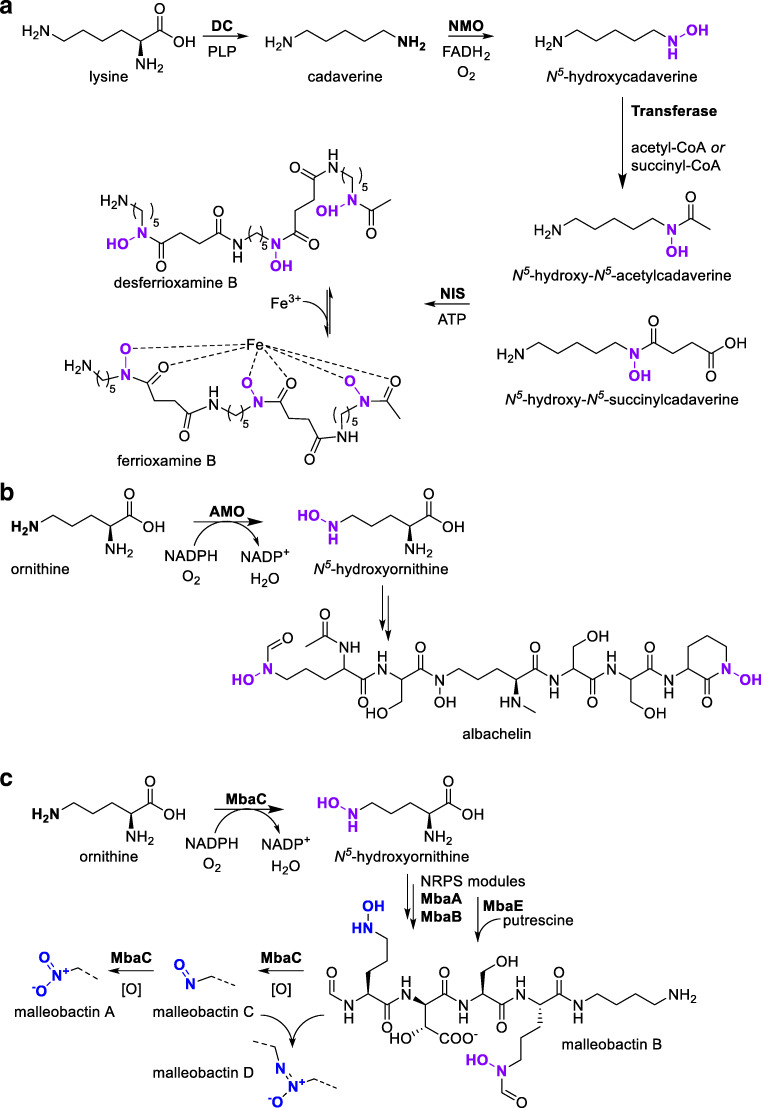
**a** Biosynthesis of desferrioxamine B and its iron chelation product ferrioxamine. **b** First, the pyridoxal phosphate (PLP)–dependent decarboxylase (DC) provides the substrate for the flavin *N*-hydroxylase (NMO), which activates molecular oxygen via reduced FAD. Then, activated acyl or succinyl residues are transferred to provide the substrate for the NRPS-independent ATP utilizing synthetase (NIS). **b** Biosynthesis of albachelin. A flavin-dependent *N*-hydroxylase (AMO) catalyzes the production of *N*^5^-hydroxyornithine, multiple subsequent steps follow. **c** Malleobactins are synthesized by the pathogenic bacterium *Burkholderia pseudomallei* in which the activity of the NMO MbaC leads to four different siderophore structures

Siderophores (greek: sidero = iron; phore = carrier) are secondary metabolites produced by many organisms (plant, bacteria, and fungi) to sequester iron in a physiological context (Kem and Butler 2015; Carroll and Moore 2018; Hofmann et al. 2020). Often stress, such as iron limitation or ecological pressure, leads to a response of producing and secreting secondary metabolites. Other related natural compounds are specific for zinc, gold, or vanadium or even unspecific with respect to the target metal or metalloid and are thus often called metallophores (Johnston et al. 2013). All these metal-chelating natural products can be synthesized by two different routes. One involves non-ribosomal peptide synthetases (NRPSs), whereas the other one is NRPS-independent and thus designated as NIS-pathway. Many secondary metabolites, including some siderophores, are synthesized through NIS-routes, which can start from simple precursors such as amino acids. The most prominent compound to be mentioned as representative for biosynthesis and application is desferrioxamine B (DFOB) (Fig. 3a). It is produced by various actinobacteria and often the major component of a metabolite cocktail of related desferrioxamines or bisucaberins secreted by the producer strain (Senges et al. 2018; Schwabe et al. 2018; Proença et al. 2019). The production starts from lysine via cadaverine as a substrate of an *N*-hydroxylating flavoprotein to yield *N*^*5*^-hydroxycadaverine. Subsequently, a substrate-relaxed acyl-transferase comes into play and produces various intermediates, which result in a variety of DFOB- or bisucaberin-like molecules via an ATP-consuming assembly line (Ronan et al. 2018).

We focus here on routes involving flavoproteins as one of the first biosynthetic steps. Enzymes studied so far are listed in Table 1. Typical precursors to bacterial siderophores are the amino acids ornithine and lysine. These and their decarboxylation products, cadaverine and putrescine, can selectively be hydroxylated by NMOs. Their mechanism of action is similar to the above example for *N*-hydroxylating ornithine monooxygenases. The resulting *N*-hydroxy-diamines and amino acids can be further acetylated by corresponding (typically formyl tetrahydrofolate-dependent) acetyltransferases to prevent overoxidation towards nitro functions, or directly used in the assembly line of siderophores, as presented for DFOB in Fig. 3a. Besides formyl tetrahydrofolate–dependent formylation (e.g., in coelichelin or rhodochelin), downstream biosynthetic routes enclose acylation or condensation by NRPS pathways towards various products.

**Table 1:** *N*-Hydroxylating flavoprotein monooxygenases (NMOs) involved in siderophore biosynthesis. Most NMOs utilize ornithine or lysine or their decarboxylation product putrescine or cadaverine as substrates;, few have been reported to use alternative substrates—all use FAD as cofactor and NAD(P)H as coenzyme

Siderophore	Organism	NMO-substrate	NMO	Accession no.	References
Ornithine *N*-hydroxylases					
Pseudobactin	*Pseudomonas* sp. B10	Ornithine	PsbA	Q9F8X0, AAG27518	Ambrosi et al. (2000)
Pyoverdine PAO	*Pseudomonas aeruginosa* PAO1	Ornithine	PvdA	Q51548, WP_003114509	Ge and Seah (2006)
Ornibactin	*Burkholderia cepacia* K56–2	Ornithine	PvdA *Bc*	O51940, AAB94515	Sokol et al. (1999)
Cupriachelin	*Cupriavidus necator* H16	Ornithine	PvdA *Re*	Q0K0K9, CAJ96465	Kurth et al. (2019)
Taiwachelin	*Cupriavidus taiwanensis*	Ornithine	TaiO	YP_002007894, WP_012356056	Kreutzer and Nett (2012)
Malleobactin	*Burkholderia pseudomallei*	Ornithine	MbaA *Bp2*	YP_001066209, WP_004535763	Alice et al. (2006)
Malleobactin A, B, C, D	*Burkholderia thailandensis*	Ornithine	MbaC	WP_011402367	Franke et al. (2013)
Ferrichrome	*Aspergillus nidulans*	Ornithine	SidA	Q7Z8P5, AAP56238	Eisendle et al. (2003)
Ferricrocin, ferrichrome	*Aspergillus fumigatus*	Ornithine	SidA	E9QYP0, AAT84594	Hissen et al. (2005); Chocklett and Sobrado (2010)
Deferriferrichrysin	*Aspergillus oryzae*	Ornithine	Dff	Q2TZB2, BAC15565	Yamada et al. (2003)
Kutznerides	*Kutzneria* sp*.* 744	Ornithine	KtzI	ABV56589	Fujimori et al. (2007)
Rhodochelin	*Rhodococcus jostii* RHA1	Ornithine	Rmo	ABG96502	Bosello et al. (2012)
Fuscachelin	*Thermobifida fusca*	Ornithine	FscE	PZN64719	Dimise et al. (2008)
Erythrochelin	*Saccharopolyspora erythraea* NRRL 2338	Ornithine	EtcB	PFG94420	Robbel et al. (2011)
Thermochelin	*Thermocrispum agreste*	Ornithine	TheA	WP_028847741	Heine et al. (2017)
Albachelin	*Amycolatopsis alba* DSM 44262	Ornithine	AMO	OXM54439	Bufkin and Sobrado (2017)
Coelichelin	*Streptomyces ambofaciens* ATCC 23877	Ornithine	CchB	A0ACR3	Pohlmann and Marahiel (2008)
Vicibactin	*Rhizobium etli* CFN 42	Ornithine	VbsO	Q2JYJ0	Heemstra et al. (2009)
Mixed substrate *N*-hydroxylases					
Aerobactin	*Shigella flexneri*	Lysine	IucD	Q9XCH1	Thariath et al. (1993)
Aerobactin	*Escherichia coli*	Lysine	Lys *Ec*	P11295	Herrero et al. (1988)
Mycobactin	*Mycobacterium smegmatis*	Lysine	MbsG	CKI16899	Robinson et al. (2014b)
Mycobactins	*Mycobacterium tuberculosis*, *Mycobacterium* sp. MCS	Lysine or acyl-lysine	MbtG	Q1B6B3	Madigan et al. (2012)
Nocobactin	*Nocardia farcinica* IFM 10152	Lysine	NbtG	BAD55606	Binda et al. (2015)
Bisucaberin	*Aliivibrio salmonicida* LFI1238	Cadaverine	BibB	CAQ81032	Kadi et al. (2008)
Desferroxamine	*Streptomyces scabiei* 87.22	Cadaverine	DesB	C9Z469	Barona-Gómez et al. (2004)
Desferrioxamine	*Erwinia amylovora* CFBP1430	Cadaverine	DfoA	D4I246, CBA23306	Salomone-Stagni et al. (2018)
Desferroxamine	*Gordonia rubripertincta* CWB2	Diamines	GorA	AOR50757	Esuola et al. (2016)
Alcaligin	*Bordetella bronchiseptica* RB50	Putrescine	AlcA	Q44740	Kang et al. (1996)
Putrabactin	*Shewanella oneidensis*	Putrescine	PubA	WP_011072933	Li et al. (2016)
Putrabactin	*Shewanella putrefaciens* 95	Putrescine	SpPMO (PubA)	QBX90611	Saroja et al. (2019)
Rhizobactin1021	*Sinorhizobium meliloti* 1021	1,3-Diaminopropane	RhbE	Q9Z3Q8	Lynch et al. (2001)

**Fig. 4:**
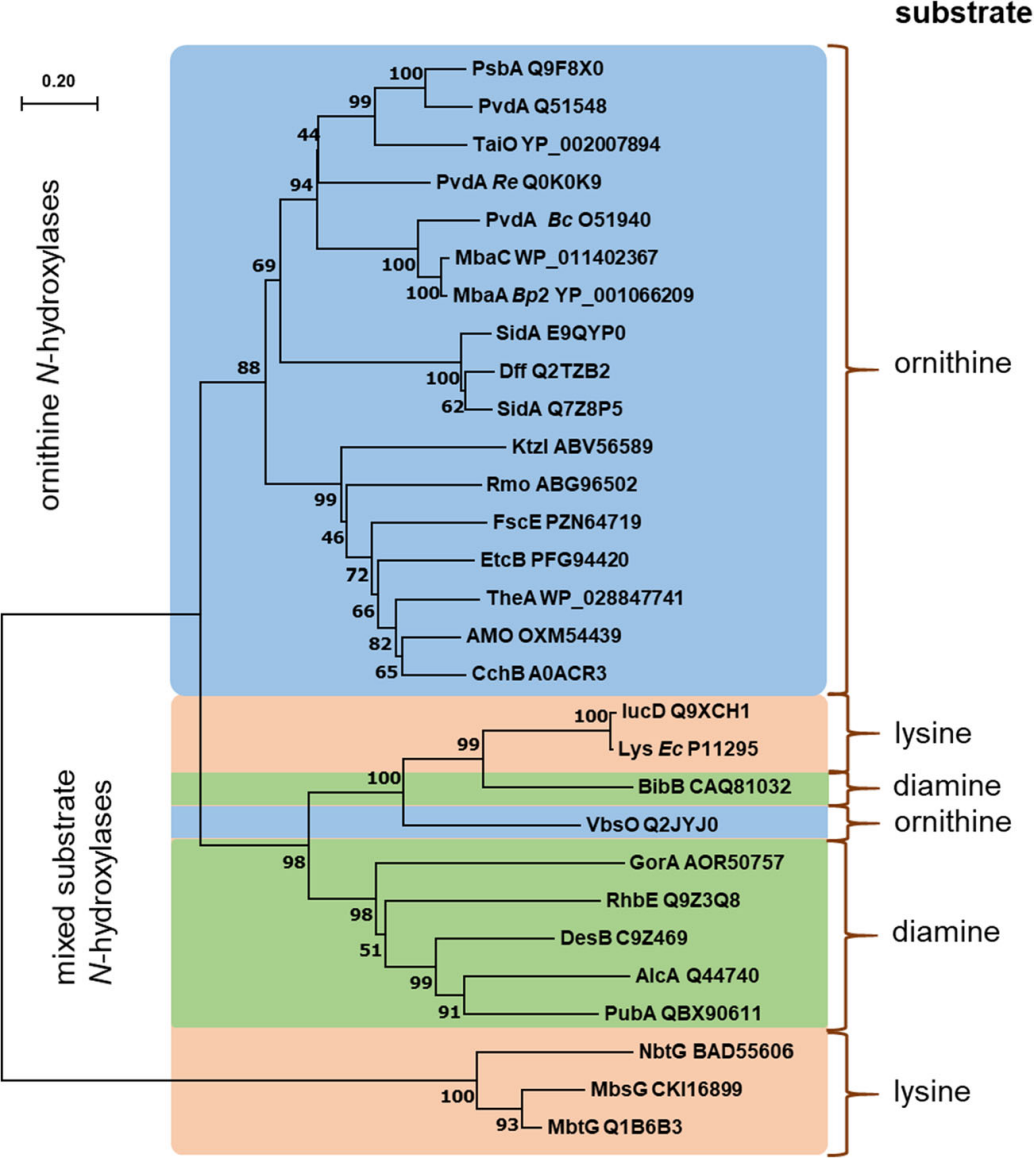
Minimum evolution distance tree of *N*-hydroxylating flavoprotein monooxygenases from bacteria and fungi in analogy to earlier studies (Franke et al. 2013; Esuola et al. 2016). Evolutionary distances were computed using the JTT matrix–based method and are given in the units of the number of amino acid substitutions per site (see scale bar). The accession numbers and protein designations are given according to Table 1 and references cited therein. It is worth to mention the most intensively studied representatives: *Aspergillus fumigatus* SidA (E9QYP0) and *Pseudomonas aeruginosa* PvdA (Q51548), respectively. The substrate of each NMO is given and respective parts of the tree are color-coded

The enzymes mentioned in Table 1 can be classified according to their amino acid sequence and clustered in phylogenetic trees as done earlier (Franke et al. 2013; Esuola et al. 2016) and presented comprehensively in Fig. 4.

On the basis of amino acid sequences, one can distinguish lysine from ornithine *N*-hydroxylases (Fig. 4). Only one enzyme with a close relation to lysine *N*-hydroxylases accepts ornithine as a substrate: VbsO (Heemstra et al. 2009). This enzyme converts solely l-ornithine, while the d-form and both lysine enantiomers yield no product. Lysine acts as a non-substrate effector of VbsO, leading to the uncoupling of hydroxylation (see also Fig. 2).

Among the lysine *N*-hydroxylases, two groups exist: one of mycobacterial and nocardia strains and another from *Escherichia coli* and related bacteria. Both are clearly separated, and the mycobacterial representatives seem most distant to all other *N*-hydroxylases. Another group of enzymes, diamine accepting *N*-hydroxylases (Table 1 and Fig. 4), seems closely related to the lysine *N*-hydroxylating NMOs from *E. coli* and related strains. Thus, we propose an evolutionary linkage that could be experimentally validated through mutagenesis studies to switch their substrate spectrum from diamine to amino acid or vice versa. Furthermore, among all NMOs, the relation is dependent on the origin of these enzymes. Thus, fungi, mycobacteria, pseudomonads, *Burkholderia* and *E. coli* (always with closely related microorganisms) form small subgroups in the phylogenetic tree.

Many enzymes share a similar pattern of activity, and the final product of metabolic pathways they are part of is typically defined by the subsequent enzymatic steps and respective substrates. Functional groups that connect NMO-based routes, often N-O bonds, are crucial for biological function. This is true for all the siderophores produced via such a route since the N-O bond is usually part of a hydroxamate functional unit that allows metal chelation. This hydroxamate unit can be located terminally as an open or ring-like structure or simply in the middle of a siderophore molecule, and several such units can be present in one single molecule.

Recently, the biosynthesis of albachelin was described (Fig. 3b) (Kodani et al. 2015). This siderophore is produced by an actinobacterium named *Amycolatopsis alba* under iron starvation. The involved NMO is designated AMO and prefers ornithine as substrate and NADPH as an electron donor. NADH does bind and allows conversion but yields only unproductive hydrogen peroxide formation. The same is true for lysine, which is only an effector and increases uncoupling as reported for other ornithine converting NMOs, such as VbsO (vide supra).

The biosynthesis of malleobactin A–D involves the NMO MbaC and leads to products with various N-O functional units such as *N*-hydroxy, *C*-nitroso, nitro, and azoxy (Fig. 3c) (Franke et al. 2013; Hedges and Ryan 2020). MbaC produces *N*^*5*^-hydroxy ornithine, which is used to assemble different malleobactins. An interesting example constitutes 2-amino-5-nitropentanoic acid, produced from ornithine as an intermediate to malleobactin A in *Burkholderia pseudomallei* (Franke et al. 2013). Interestingly in this case, the formation of a terminal nitro group is made possible, even though this oxidation would typically be circumvented in other metabolic pathways by immediate acetylation of the formed *N*-hydroxy group, thereby also creating a bidentate ligand for metal chelation. It is postulated that the NMO in this case does support the conversion of *N*-hydroxy group towards the nitro group (Fig. 4b) (Franke et al. 2013).

The diverse nature of siderophores and their structural as well as functional elements provide access towards many applications, as has been extensively reviewed (Saha et al. 2016; Su et al. 2018; Albelda-Berenguer et al. 2019; Řezanka et al. 2019; Hofmann et al. 2020) and will not be discussed in detail here.

## NMOs with structurally complex substrates

While flavin-based NMOs related to siderophore biosynthesis typically employ the same “simple” substrates to build up highly complex structures, other NMOs have been disclosed that are able to employ structurally quite diverse substrates, ranging from small nonproteinogenic amino acids to large and complex units. The investigation of these enzymes can reveal valuable insights for future biotechnological applications in hitherto unexploited fields.

### NMOs for diverse bacterial substrates

**Fig. 5:**
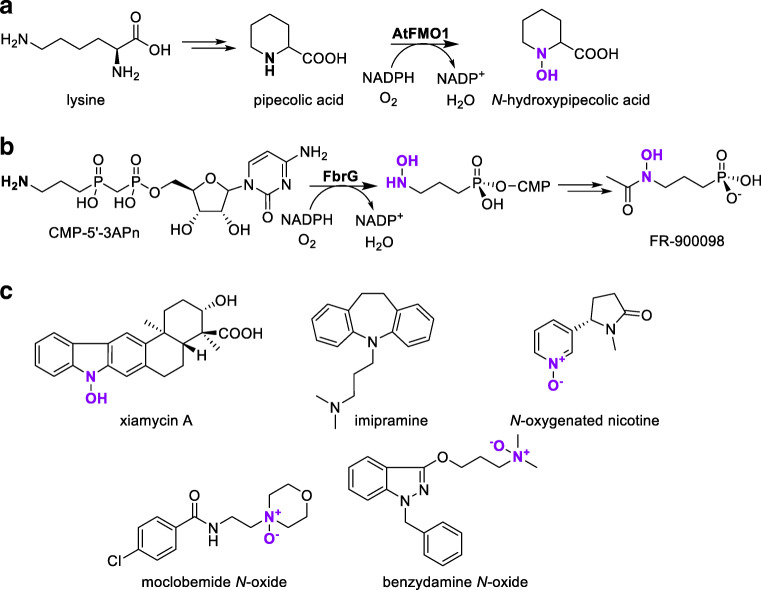
**a***At*FMO1-catalyzed hydroxylation of pipecolic acid to *N*-hydroxypipecolic acid, which provides systemic acquired resistance for plants. **b** FrbG-catalyzed hydroxylation of the *N*-acetyl-3-aminopropylphosphonate in the biosynthetic pathway towards the antimalarial agent FR-900098. **c** Xiamycin A (XMA) is *N*-hydroxylated by XiaK. hFMO1 catalyzes the *N*-oxygenation of imipramine, whereas hFMO3 catalyzes the *N-*oxygenation of nicotine as well as of moclobemide to produce the human drug metabolite moclobemide-*N*-oxide. *N*-oxygenation of the anti-inflammatory drug benzydamine by a flavin-containing monooxygenase located in the lungs produces benzydamine-*N*-oxide

A flavin-dependent NMO named *At*FMO1 from the plant *Arabidopsis thaliana* hydroxylates pipecolic acid to *N*-hydroxypipecolic acid (Hartmann and Zeier 2018; Hartmann et al. 2018). This reaction is part of a pathogen-inducible catabolic pathway of lysine that has a central function in systemic acquired resistance (Fig. 5a).

Recently, the groups of Zhao and Nair disclosed the crystal structure and characterization of an FAD-dependent NMO from *Streptomyces rubellomurinus*, FrbG, that catalyzes the hydroxylation of an aminopropylphosphonate unit within a more complex CMP conjugate (Nguyen et al. 2019). FrbG shares structural similarities with group B FMOs and takes part in the biosynthesis of the antimalarial agent FR-900098, which requires a hydroxamate (Fig. 5b).

The structure obtained from crystallization of FrbG contains an FAD prosthetic group and NADPH coenzyme co-crystallized together, displaying a proper orientation of the nicotinamide ring stacking with the flavin isoalloxazine moiety for optimal hydride transfer. Contrary to group B FMOs (Fig. 2b), the NADPH-binding domain also confers substrate recognition, with the cytidine-5′-monophosphate moiety being crucial for substrate specificity. A conformational reorganization likely occurs after flavin reduction by NADPH (Nguyen et al. 2019), similar to the “moonlighting” effect observed with group B FMOs, i.e., their ability to take over more than one function (Alfieri et al. 2008). The discovery of new types of *N*-hydroxylases such as FrbG through cloning and sequencing of biosynthetic gene clusters paves the way for metabolite synthesis.

Another bacterial NMO called XiaK, identified from the biosynthetic gene cluster of the indolosesquiterpene xiamycin A (XMA) from a *Streptomyces* strain, catalyzes the hydroxylation of XMA (Fig. 5c). The groups of Zhang and Liu confirmed XiaK to be a flavin-dependent enzyme functioning as an *N*-hydroxylase in the biosynthesis of indolosesquiterpene intermediates (Zhang et al. 2017). Recombinant expression and characterization of XiaK showed that it only catalyzed the hydroxylation of XMA and that the *N*-hydroxylated product decomposed to other compounds in a possible enzyme free radical–mediated mechanism. This highlights the possibility that isolated compounds from microorganisms may not be “true” secondary metabolites but non-enzymatic derivative products of a biosynthesized compound.

### FMOs from human/mammalian cells with NMO activity

The human family of FMOs includes five known enzymes, hFMO1–5. As already introduced above, these FMOs belong to the same group of flavoprotein monooxygenases and are able to oxygenate carbon-linked heteroatoms (van Berkel et al. 2006). The five hFMO enzymes exhibit a tissue-specific expression pattern in adults (Perez-Paramo et al. 2019): hFMO1 is expressed in kidneys and hFMO3–5 in the liver, whereas hFMO2 is mainly expressed in the lungs. hFMO5, which was shown to catalyze Baeyer-Villiger oxidation reactions (Fiorentini et al. 2016; Fiorentini et al. 2017), is now commercially available (Gecco Biotech B.V.).

Recently, hFMO3 was reported to exhibit activity towards nicotine (Fig. 5c) (Perez-Paramo et al. 2019). The substrate versatility of this enzyme makes it an attractive catalyst for future applications with complex substrates and towards the synthesis of drug metabolites. Other hFMOs are also involved in nicotine detoxification processes through *N-*oxygenation in CYP2A6-deficient humans, with hFMO1-3 displaying higher activity and hFMO4-5 lower activity (Perez-Paramo et al. 2019). Furthermore, hFMO1 catalyzes the *N*-oxygenation of imipramine, among others (Fig. 5c) (Furnes and Schlenk 2004).

A drawback of using hFMOs in biocatalytic applications is their low production levels using recombinant expression in *Escherichia coli*. Nevertheless, Hanlon et al. managed to produce a sufficient amount of hFMO3 for preparative biotransformation with a large-scale cultivation of recombinant *E. coli* harboring the gene encoding for hFMO3 (Hanlon et al. 2012). In this way, the authors were able to produce milligram amounts of the drug metabolite moclobemide-*N*-oxide (Fig. 5c). Such distinctly produced drug metabolites can e.g. be used in the detailed study of metabolites’ mode of action, among others.

Another example is the anti-inflammatory drug benzydamine, metabolized by an FMO located in the lungs, possibly hFMO2 (Fig. 5c) (Störmer et al. 2000). Benzydamine was recently used as a substrate to observe pulmonary FMO activity in rats to assess its metabolic fate (Yilmaz et al. 2019).

## NMOs promoting the formation of N-N bonds

N-N bonds are abundant in countless biological compounds (Blair and Sperry 2013; Waldman et al. 2017). Diazo compounds especially have versatile biological functions and are present in numerous biological systems, as has recently been extensively reviewed (Nawrat and Moody 2011; Mix et al. 2016). There are many ways of producing N-N and N=N bonds; NMOs are involved in some of them in different manners. It is especially interesting to see that the biocatalytic pathways of N-N bond containing biomolecules, and thus, the involved enzymes have only been scarcely explored in the past and are gaining increasing attention in recent literature. General pathways have been explored, leading to biomolecules with diverse structures and functions, of which a few are highlighted here.

### Amino acid *N*-hydroxylation and subsequent nitrous acid production

**Fig. 6:**
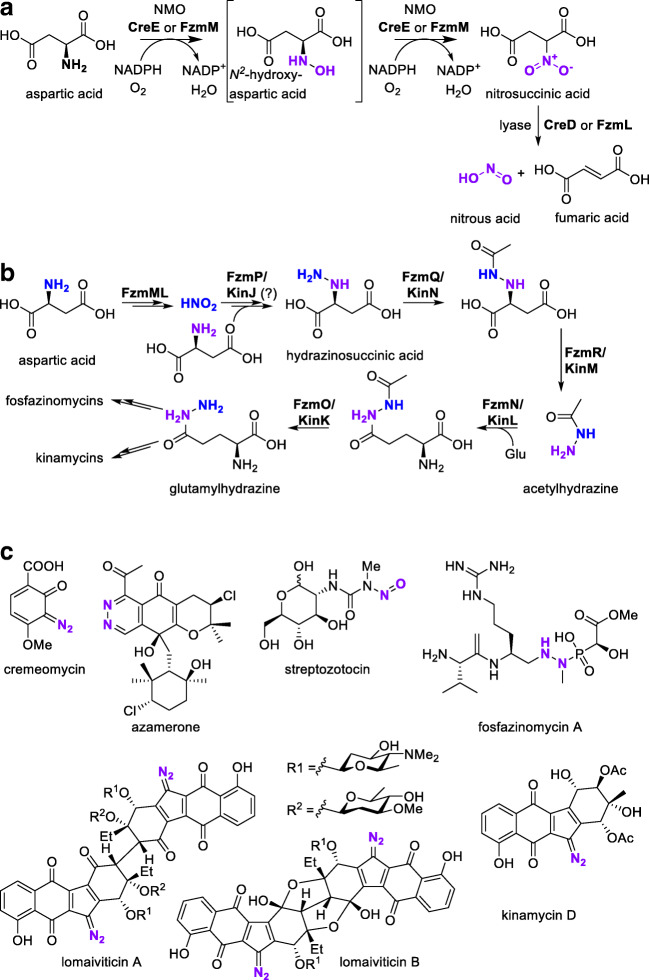
**a** Release of nitrous acid from aspartic acid, promoted by a double hydroxylation and lyase reaction. **b** Biosynthesis of fosfazinomycins and kinamycins via the same initial intermediates. **c** Examples of biomolecules for which biosynthesis involves the NMO-driven generation of nitrous acid

One of the most prominent ways in which NMOs are involved in the generation of N-N bonds is by liberation of nitrous acid starting from the *N*^*2*^-hydroxylation of aspartic acid (Fig. 6a). A stepwise over-oxidation of the amino group to a nitro function by a flavin-based NMO results in the formation of nitrosuccinic acid via the instable *N*^2^-hydroxyaspartic acid intermediate (Wang et al. 2018). The oxidation step is followed by a lyase reaction, resulting in cleavage of the nitro group and producing fumaric acid as a second product. The liberated nitrous acid is discussed to react with a primary amine of a second substrate molecule in a non-enzymatic way, thus forming a hydrazine-like intermediate with an N-N bond which can be further modified to produce manifold products. Both the NMO and the lyase are well conserved and widespread in different organisms. It should be mentioned that not only flavin-containing NMOs are capable of liberating small NO compounds; there is a variety of biosynthetic processes that involve “free” NO throughout organisms (Caranto 2019). Several nitrous acid-derived products have recently been reported involving this general biosynthetic pathway, most of them having potential activities as antibiotics or even anti-proliferative agents (Fig. 6c, Table 2, section A).

**Table 2:** Reported compounds for which the biosynthesis involves either (A) an NMO-induced nitrous acid release or (B) an NMO-catalyzed *N*-hydroxylation and subsequent condensation reaction to generate (natural) products with complex functional groups

Compound	Organism	NMO	Additional reaction remarks		References
**A. NMO-induced nitrous acid release**					
			**Further relevant enzymes**	**Coupling partner for HNO**_**2**_	
**Cremeomycin**	*Actinomyces cremeus* NRRL3241	CreE	CreD (lyase)	3-Amino-2-hydroxy-4-methoxybenzoate	Waldman et al. (2015); Sugai et al. (2016)
**Fosfazinomycin**	*Streptomyces* sp. NRRL S-149	FzmM	FzmL	Aspartate	Gao et al. (2014); Huang et al. (2016)
**Kinamycin**	*Streptomyces murayamaensis*	FzmM	FzmL	Aspartate	Wang et al. (2018)
**Lomaiviticin**	*Salinispora* sp.	Strop2198			Kersten et al. (2013)
**Azamerone**	*Streptomyces* sp. CNQ-766	n.d.	SF2415B1 after C-amination	Amino dihydroquinone intermediate	Winter et al. (2009)
**Streptozotocin**	*Streptomyces achromogenes*	Unknown		citrulline derivative	Le Goff and Ouazzani (2014)
**Triacsins**	*Streptomyces aureofaciens* ATCC31442	Tri21 (Asp hydroxylase)	Tri16 (lyase)	Hydrazinoacetate derivative	Twigg et al. (2019)
**Alanosine**	*Streptomyces alanosinicus* DSM 40606	AlnM	AlnN	(Diaminopropionate bound to AlnI via PCP)	Wang et al. (2020)
**B. NMO-catalyzed*N*-hydroxylation and subsequent condensation reactions**					
			**Substrate**	**N-OH condensates with**	
**Piperazic acid**	Actinomycete bacteria	PzbA homologs	Ornithine (*ω* position)	Itself (*α* position)	Hu et al. (2019)
**Kutznerine**	*Kutzneria* sp. 744	KtzI	Ornithine (*ω* position)	Itself (*α* position)	Fujimori et al. (2007)
**S56-p1 (hydrazinoacetic acid)**	*Streptomyces* sp. SoC090715LN-17	Spb38	Lysine	Glycine	Matsuda et al. (2018)
**Valanymicin**	*Streptomyces Viridifaciens* MG456-hF10	VlmH	Isobutylamine	Serine	Parry and Li (1997); Tao et al. (2003); Garg et al. (2008); Garg et al. (2009)
**Pyridazomycin**	*Streptomyces violaceoniger* sp. *griseofuscus*	Unknown	Ornithine	Oxaloacetate	Bockholt et al. (1994)
**Triacsins**	*Streptomyces aureofaciens* ATCC31442	Tri26	Lysine	Glycine	Twigg et al. (2019)
**Alanosine**	*Streptomyces alanosinicus* DSM 40606	AlnG	l-Diaminopropionate bound to AlnI via PCP	NO_*x*_	Wang et al. (2020)

Cremeomycin (Fig. 6c) is one of the best-investigated examples of an antibiotic with a diazo function (Waldman et al. 2015; Sugai et al. 2016). Initially isolated from *Actinomyces cremeus* (NRRL3241), it adversely affects Gram-positive and Gram-negative bacteria and has antifungal and even antitumor activities (Bergy and Pyke 1967). The diazo-containing moiety of cremeomycin is built up by a late-stage diazotization of an amino function by nitrous acid, itself generated by the typical cascade: aspartic acid is hydroxylated/over-oxidized by the NMO CreE; the formed nitrosuccinic acid liberates nitrous acid by aid of the lyase CreD (Waldman and Balskus 2018).

Fosfazinomycins and kinamycins (Fig. 6b, c) are two antibiotic classes produced by and initially isolated from *Streptomyces* strains (*S. lavendofoliae* 630 and *S. murayamaensis*, respectively) (Ito et al. 1970; Kuroda et al. 1980). Despite their structural diversity, they share the same initial steps of biosynthesis (Fig. 6b) (Wang et al. 2018). Aspartate is hydroxylated by the NMO FzmM to nitrosuccinate in two oxidative stages; liberation of nitrous acid is catalyzed by FzmL, a 3-carboxymuconate cycloisomerase. In multiple subsequent enzymatic steps, the central intermediate glutamylhydrazine is generated via hydrazinosuccinate and acetylhydrazine (Huang et al. 2016). The identified FzmM shows homology to an FAD(NAD)-dependent oxidoreductase from *Streptomyces davawensis* JCM 4913 (WP_015660731; 56% amino acid identity, 65% similarity) (Gao et al. 2014).

Lomaiviticins (Fig. 6c) are structurally similar to kinamycins, making their biosynthetic origin very likely similar. They are regarded as highly potent antitumor active agents and were initially isolated from *Salinispora pacifica* (Mix et al. 2016). A potential gene cluster for lovamaiviticin biosynthesis was found in *Salinispora tropica* CNB-440. The gene *strop2198* was assumed to code for a putative FAD-dependent monooxygenase based on homology to *Streptomyces albaduncus* JagF (CBH32087; 61% amino acid identity 52% similarity) (Kersten et al. 2013).

Azamerone (Fig. 6c) is another compound with an uncommon N-N bond. The potential topoisomerase inhibitor was first isolated from *actinomycetes* MAR4 (Cho et al. 2006). The biosynthesis of azamerone was initially believed to occur homologous to pyridazomycin (cf. below), but isotope labeling studies indicated that the origin of the diazo function in confirmed precursors to the compound stems from nitric acid, therefore involving the *N*^2^-hydroxylation of aspartic acid by an NMO (Winter et al. 2009).

In the case of streptozotocin (Fig. 6C), an anticancer antibiotic known since the 1950s, it is also believed that the nitrosamine functional group originates from a central nitrous acid as intermediate. However, the biocatalytic pathway has not been elucidated to date (Le Goff and Ouazzani 2014).

### *N*-Hydroxylation and condensation with amino or hydroxy functions

The second typical way to generate N-N bonds in biosynthesis with the aid of flavin-NMOs is the condensation of an NMO-generated hydroxylamine with carboxy functions. Subsequent rearrangement steps can result in new N-N bond containing molecules (Table 2, section B).

**Fig. 7:**
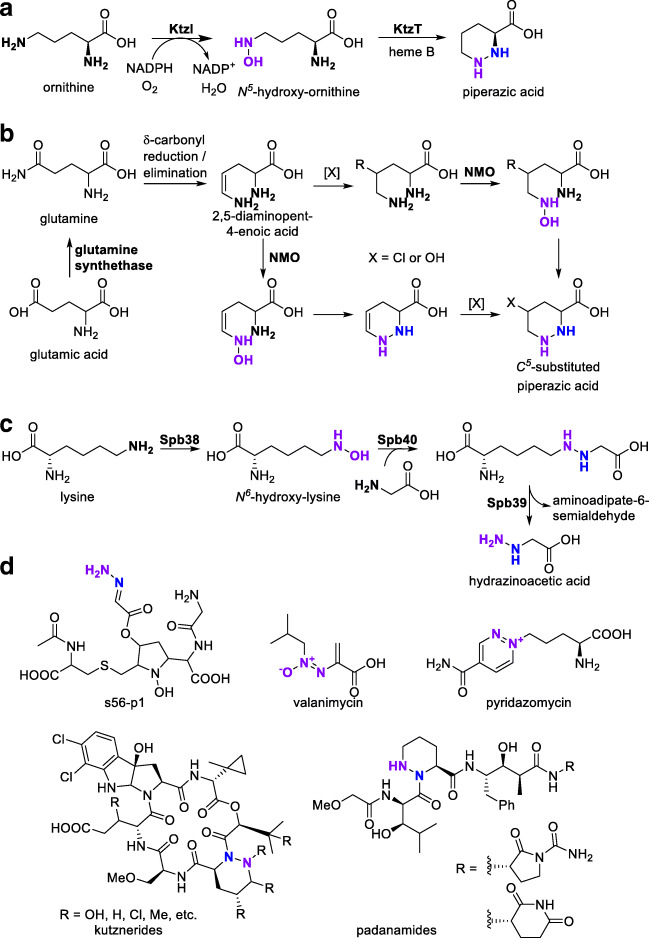
**a** Biosynthesis of piperazic acid. **b** Biosynthesis of substituted piperazic acids via two proposed pathways. **c** Biosynthesis of hydrazinoacetic acid as a building block for more complex biomolecules. **d** Diverse N-N bond containing biomolecules: s56-p1 is derived from hydrazinoacetic acid, kuznerides, and padanamides contain piperazic acid building blocks. Valanimycin and pyridazomycin are further recently investigated examples

Piperazic acid is one of the most common N-N containing building blocks of natural products, being found in numerous natural compounds with activities ranging from antibiotics to immunosuppressants. All piperazic acid-containing molecules known to date come from Actinomycete bacteria isolated from diverse environments (Morgan et al. 2019), but the structural and functional variety is remarkable: kutznerides, padanamides, himastatins, monamycines, polyoxypeptin and sanglifehrins, to name a few, contain piperazic acid (Fig. 7a). A recent review demonstrates the versatile incorporation of piperazic acid into natural products (Morgan et al. 2019).

The synthesis of piperazic acid starts with the *N*^*5*^-hydroxylation of ornithine by a flavin-based NMO (Fig. 7a) (Neumann et al. 2012). *N*^5^-hydroxyornithine itself does not cyclize spontaneously (Le Goff and Ouazzani 2014). Instead, a heme-dependent protein, typically termed piperazate synthase, fuses the two nitrogen atoms in a condensation reaction, creating piperazic acid (Du et al. 2017). The enzymatic pair is highly conserved in the gene clusters responsible for the synthesis of different piperazic acid–containing molecules (Morgan et al. 2019).

Depending on the final product, the enzyme nomenclature varies. The best-described case, with a detailed functional and structural investigation of the flavin-based NMO, is probably the production of kutznerine (Fig. 7d) by *Kutzneria* sp. 744, where the two enzymes are designated as *N*-hydroxylase KtzI and piperazate synthase KtzT (Neumann et al. 2012; Setser et al. 2014; Du et al. 2017). A recent bioinformatic study revealed, next to numerous stand-alone enzyme pairs, the existence of 11 chimeric two-enzyme pairs with unique position-specific amino acid utilization patterns compared with the stand-alone homologs (Hu et al. 2019).

Furthermore, derivatives of piperazic acid, such as 5-hydroxy-, 5-chloro-, and dehydro-piperazic acid can be produced and incorporated into more complex structures such as piperazimycins. There are two theories about how these derivatives are produced from glutamic acid (Fig. 7b) (Miller et al. 2007): the central intermediate 2,5-diaminopent-4-enoic acid is hydroxylated at the *N*^*5*^ position and, subsequently, addition of a heteroatom to the double bond occurs. Alternatively, *N-*hydroxylation takes place after the addition of the heteroatom to the double bond (Oelke et al. 2011; Handy and Sello 2017). Alternatively, downstream modification of piperazic acid has been discussed after it has been synthesized from ornithine in its cyclic form (Jiang et al. 2011).

Hydrazinoacetic acid is another building block which has been reported in different biosynthetic pathways. It is proposed to be an intermediate in the biosynthesis of a complex natural compound s56-p1, isolated from *Streptomyces lividans* through heterologous expression (Fig. 7c)*.* It is built from lysine, which is transformed into *N*^*6*^-hydroxylysine by the flavin-dependent hydroxylase Spb38, followed by condensation with glycine through Spb40. The intermediate hydrazine adduct is then oxidized at the C^6^-N bond by Spb39, which was identified as flavin-dependent d-amino acid oxidase homolog. This reaction results in liberation of hydrazinoacetic acid, which is expected to be directly incorporated into the target compound s56-p1 (Matsuda et al. 2018). The gene cluster was identified and annotated based on homology annotation, with the closest homology for all three genes originating from *Catenulispora acidiphila* DSM 44928 (Sbp38: 85% amino acid identity, 90% similarity to the respective lysine monooxygenase) (Matsuda et al. 2017).

For valanomycin (Fig. 7d), a versatile azoxy compound from *Streptomyces viridifaciens* MG456-hF10, biosynthesis involves the decarboxylation of valine and *N*-hydroxylation of the intermediate isobutylamine. Based on functional and homology annotation, the VlmH/R enzyme pair was denoted as FAD/NADPH-dependent isobutylamine hydroxylase and FAD reductase, respectively. Here, *vlmH* encodes for an enzyme of 378 amino acids homologous to an isobutylamine *N*-hydroxylase from *Streptomyces avermitilis* (BAB69230, 51% amino acid identity and 67% similarity) (Garg et al. 2002). In subsequent steps to generate the -N^+^(O^−^)=N- unit, different mechanisms have initially been proposed for a reaction of isobutylhydroxylamine with serine (Tao et al. 2003; Garg et al. 2008; Garg et al. 2009); the condensation of the N-OH function with the serine-carboxy function and successive rearrangement has been widely accepted in the literature (Le Goff and Ouazzani 2014).

The biosynthesis of pyridazomycin (Fig. 7d) is not well understood, but it is believed that pyridazomycin originates from ornithine, oxaloacetate, and glycine, making the intermediate formation of *N*^5^-hydroxyornithine which condensates with oxalacetate a very probable hypothesis (Bockholt et al. 1994; Wermuth 2011).

## Compounds with more than one NMO involved in biosynthesis

**Fig. 8:**
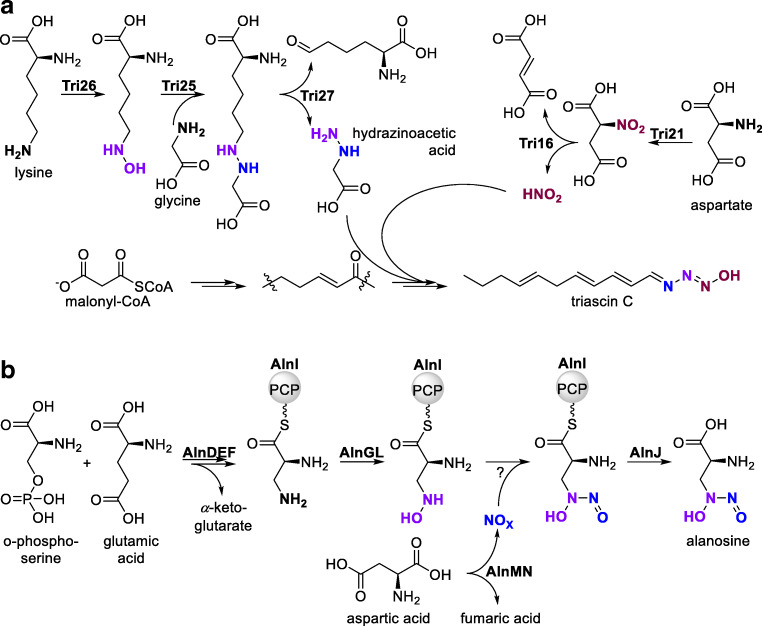
**a** The biosynthesis of triacsins involves two *N*-hydroxylation steps catalyzed by two different NMOs. **b** Two *N*-hydroxylase-catalyzed reaction steps are also involved in the biosynthesis of alanosine

Triacsins are another class of compounds with highly interesting properties and peculiar biosynthetic origin first isolated from *Streptomyces aureofaciens* ATCC 31442 (Twigg et al. 2019). The compounds bear an *N*-hydroxytriazene unit terminally bound to a poly-unsaturated C11 alkyl chain (Fig. 8a). The compounds therefore structurally mimic fatty acids and bear multifaceted biological functions, ranging from acetyl-CoA-synthetase inhibition to antimalarial and antiviral activities. For the biosynthesis of the *N*-hydroxytriazene functional group, two independent *N*-hydroxylation steps are required (Fig. 8a). Using [^15^N] isotope labeling and homology annotation, the enzyme Tri21 was recently identified as a flavin-dependent NMO, putatively catalyzing aspartate overoxidation finally leading to nitrous acid liberation (Twigg et al. 2019). The second *N*-hydroxylation step was ascribed to Tri26, a putative lysine monooxygenase. Subsequent condensation of the *N*^6^-hydroxy function with glycine produces first an internal hydrazine unit and next hydrazinoacetic acid. The fatty acid backbone and the two nitrogen components are then assembled in multiple, not yet fully understood steps. Both NMOs involved in this pathway were annotated as flavin-dependent *N*-hydroxylases, with Tri21 showing homology to an FAD/NAD(P) binding protein from *Salinispora pacifica* (WP_018723641; 67% amino acid identity, 75% similarity) and Tri26 being homologous to *N*-hydroxylase MbtG in *Streptomyces ipomoeae* (WP_009311506, 81% amino acid identity, 89% similarity) (Krithika et al. 2006).

Another important intermediate in the biosynthesis of more complex structures is the non-proteinogenic amino acid alanosine, itself discussed to have antibiotic, antiviral and anticancer activities (Fig. 8b) (Ng et al. 2019). Its biosynthesis was elucidated from the alanosine pathway in *Streptomyces alanosinicus* sp. (Ng et al. 2019; Wang et al. 2020). A gene pair *alnM/N* was identified with homology to the above-described *creD/E* and *fzmM/N* pairs, with AlnM being annotated as aspartate-converting amine hydroxylase, eventually promoting the liberation of NO_2_^−^. The gene *alnM* was found as 76% identity homolog to a similar gene from *Streptomyces kanamyceticus* (WP_055544225). Interestingly, a second *N*-hydroxylation step is discussed within the biosynthesis pathway involving enzymes denoted as AlnG and AlnL. The former was described as a putative flavin-dependent acyl-CoA dehydrogenase, which operates in the oxidative direction to activate oxygen for *N*^4^-hydroxylation of a diaminopropane unit (Wang et al. 2020). A*lnG* was found as 89% identity homolog to a gene from *Streptomyces hirstus* (WP_055594947). Overall, 14 gene clusters with relation to the l-alanosine gene cluster were identified in *Streptomyces* and *Saccharothrix* sp., all containing genes similar to *alnG* but none with genes for the *alnM/N* pair (Wang et al. 2020).

## Perspectives

In this review, we focused on the remarkable ability of flavin-dependent enzymes to hydroxylate nitrogen-containing compounds, leading to a myriad of possible functional groups which play a major role in the bioactivity of secondary metabolites. The detailed understanding of ornithine-converting NMOs and their mode of action has set the basis for focused research efforts to include this enzyme class in biocatalytic processes. These efforts have especially stimulated processes employing NMOs from the siderophore biosynthetic pathways.

Here, we highlighted the versatility in substrate scope some recently divulged NMOs can have and showcased their broad occurrence in different biosynthetic surroundings. It is especially noteworthy that many of these NMOs have just been discovered from genomics and/or proteomics studies and that they have not been characterized in an enzymological or biocatalytic context. We thus see great potential in these recent developments: From substrate scope to applications, flavin-dependent NMOs are key enzymes that we expect to play an even more prominent role in biotechnology in the near future.
